# COVID-19 and HIV infection co-pandemics and their impact: a review of the literature

**DOI:** 10.1186/s12981-021-00335-1

**Published:** 2021-05-05

**Authors:** Sivaporn Gatechompol, Anchalee Avihingsanon, Opass Putcharoen, Kiat Ruxrungtham, Daniel R. Kuritzkes

**Affiliations:** 1 HIV-NAT, Thai Red Cross AIDS Research Centre, Bangkok, Thailand; 2 Tuberculosis Research Unit, Faculty of Medicine, Chulalongkorn University, Bangkok, Thailand; 3 Division of Infectious Disease, Department of Medicine, Faculty of Medicine, Chulalongkorn University, Bangkok, Thailand; 4 Chula Vaccine Research Center (ChulaVRC)Faculty of Medicine, Chulalongkorn University, Bangkok, Thailand; 5 Division of Infectious Diseases, Brigham and Women’s Hospital, Harvard Medical School, Boston, MA USA

**Keywords:** COVID-19, HIV, Co-infection, Pathophysiology, Clinical, Outcome

## Abstract

Coronavirus disease 2019 (COVID-19) was first detected in December 2019. In March 2020, the World Health Organization declared COVID-19 a pandemic. People with underlying medical conditions may be at greater risk of infection and experience complications from COVID-19. COVID-19 has the potential to affect People living with HIV (PLWH) in various ways, including be increased risk of COVID-19 acquisition and interruptions of HIV treatment and care. The purpose of this review article is to evaluate the impact of COVID-19 among PLWH. The contents focus on 4 topics: (1) the pathophysiology and host immune response of people infected with both SARS-CoV-2 and HIV, (2) present the clinical manifestations and treatment outcomes of persons with co-infection, (3) assess the impact of antiretroviral HIV drugs among PLWH infected with COVID-19 and (4) evaluate the impact of the COVID-19 pandemic on HIV services.

## Introduction

In December 2019, a cluster of cases of pneumonia was reported in Wuhan, Hubei Province, China. The source of the infection was later identified as severe acute respiratory syndrome coronavirus 2 (SARS-CoV-2), which causes the coronavirus disease 2019 (COVID-19). On March 11, 2020 the World Health Organization (WHO) declared COVID-19 as a global pandemic [1]. As of 22 March 2021, more than 123 million cases have been confirmed globally along with 2.7 million deaths [2]. Older age and comorbidities such as hypertension, diabetes and cardiovascular disease are risk factors for developing severe COVID-19 and are associated with a high mortality rate [3–5]. However, data are limited on the impact of COVID-19 on people living with HIV (PLWH) who may be considered an immunocompromised population. Globally, there are 38 million PLWH, 690,000 of whom died of HIV-related illnesses in 2019 [6]. The WHO estimated that only 67% of PLWH were on antiretroviral therapy (ART) worldwide in 2019 [7]; only 60% of the people with HIV infection were aware of their HIV status [8]. Therefore, PLWH not on ART or whose disease is not well controlled may be at increased risk for contracting COVID-19 because their immune systems are compromised. Such persons may also be at increased risk for developing serious symptoms and death if infected with COVID-19. Limited information is available, however, regarding the treatment and outcome of SARS-CoV-2 infection among PLWH.

Recently, many countries have reported disruptions in the delivery of HIV services and care due to the COVID-19 pandemic [9]. This disruption may increase the morbidity and mortality among PWLH beyond those infected with COVID-19 [10, 11]. However, the information regarding the impact of COVID-19 on HIV services is scarce.

In this review, we describe the pathophysiology and host immune response of people infected with both SARS-CoV-2 and HIV, present the clinical manifestations and treatment outcomes of persons with co-infection, assess the impact of antiretroviral HIV drugs among PLWH infected with COVID-19 and evaluate the impact of the COVID-19 pandemic on HIV services.

## Pathophysiology and host immune response to SARS-CoV-2 and HIV infections

The pathophysiology and immune response of people infected with COVID-19 has been reviewed [12, 13]. In brief, SARS-CoV-2 is a β coronavirus characterized by its four structural proteins: Spike (S), membrane (M), envelop (E) and nucleocapsid (N) [14]. The life cycle of SARS-CoV-2 consists of the following 5 steps: attachment, penetration, biosynthesis, maturation and release [12]. Angiotensin converting enzyme 2 (ACE2) is the receptor for SARS-CoV-2 [15, 16]. Single-cell RNA sequencing has shown that ACE2 is highly expressed in the lung, heart, ileum, kidney and bladder [17]. In addition, the acute loss of smell (anosmia) has been reported in patients with COVID-19 [18]. However, ACE2 was not detected in either olfactory sensory neurons or olfactory bulb neurons [19]. Therefore, infection of non-neuronal cell types may be primary source leads to olfactory dysfunction in patients with COVID-19.

**Fig. 1:**
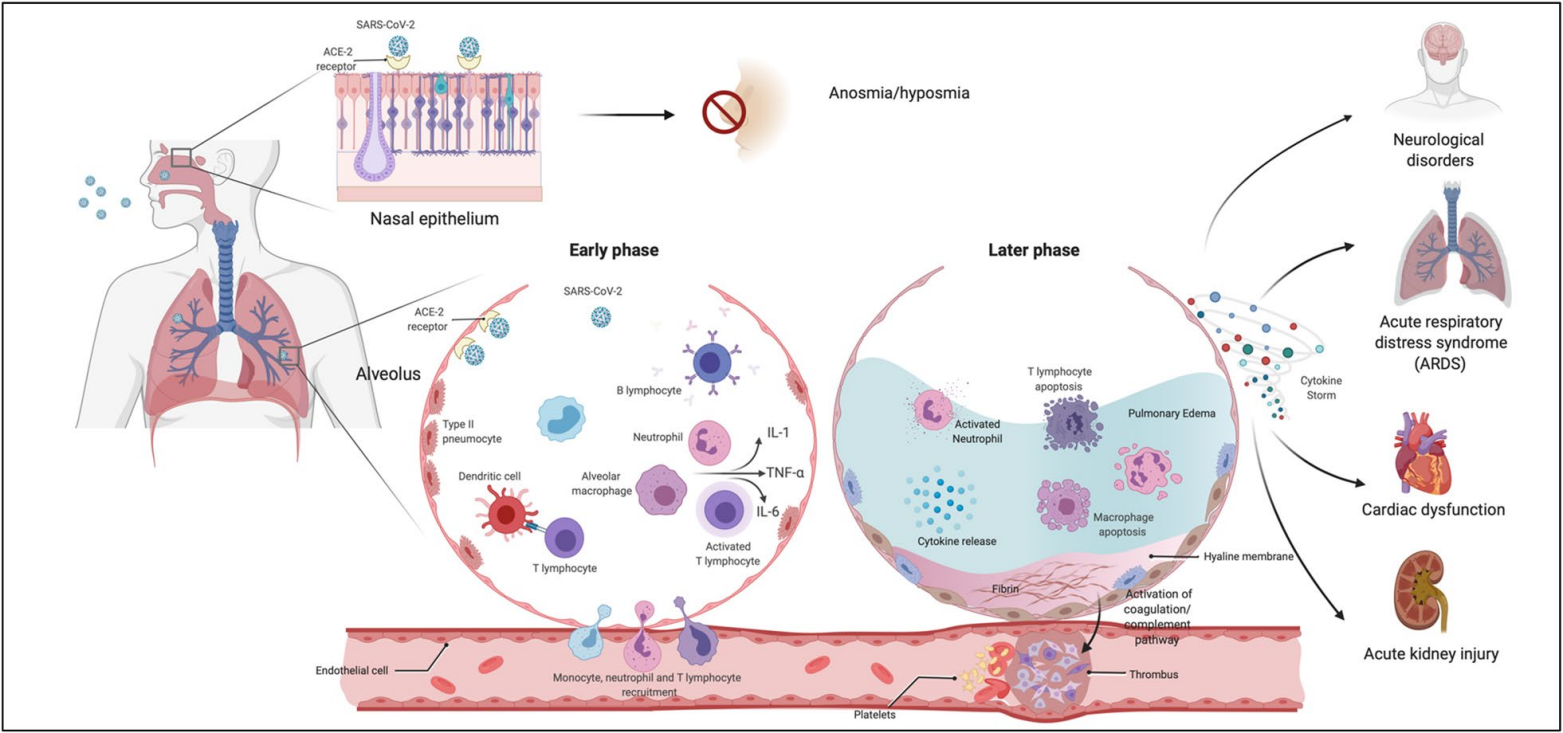
Pathophysiology and clinical manifestations of COVID-19. Created with BioRender.com

After SARS-CoV-2 infects epithelial cells of the upper respiratory tract, virus produced by infected cells travels to the lower airway, infecting bronchial and alveolar epithelial cells and alveolar macrophages [20]. As a consequence of innate immunity, virus-infected epithelial cells undergo apoptosis and are phagocytized by antigen presenting cells (APC) such as dendritic cells (DCs) and macrophages. The APC migrate to draining lymph nodes to present viral antigens to T cells [12]. Both CD4+ and CD8+ T cells play a major role in fighting against the coronavirus [21]. CD4+ T cells activate B cells to promote the production of virus-specific antibodies, while CD8+ T cells can directly kill virally infected cells [12]. In severe COVID-19, interstitial mononuclear inflammatory infiltrates, dominated by lymphocytes, are seen in lung biopsy [22]. Furthermore, desquamation of pneumocytes and pulmonary oedema with hyaline membrane formation indicating acute respiratory distress syndrome (ARDS) have been reported [22, 23]. A summary of the pathophysiology and clinical manifestations of COVID-19 are shown in Fig. 1.

**Fig. 2:**
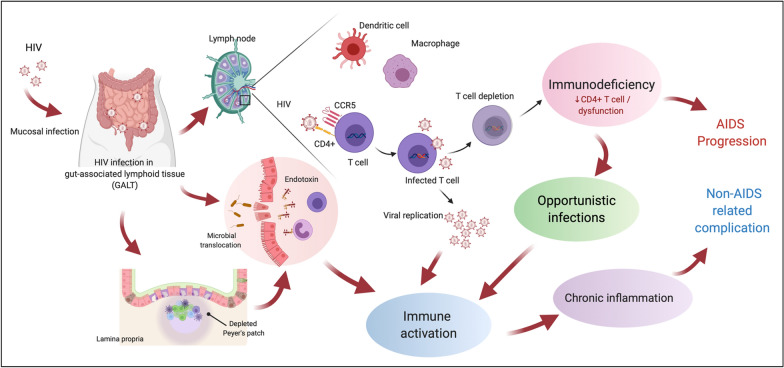
Host immune response following exposure to HIV. Created with BioRender.com

In contrast, HIV binds to the CD4 receptor of the host cell, followed by engagement of a co-receptor (i.e., chemokine receptor 5 (CCR5) or chemokine receptor 4 (CXCR4)). The characteristics of SARS-CoV-2 are compared to HIV in Table 1. All CD4 positive cells (i.e., T helper cells), macrophages, dendritic cells and astrocytes are susceptible to HIV [24]. Direct viral cytopathic effects of HIV as well as indirect effects including host innate immune response to viral DNA produced during abortive infection and endotoxin/microbial translocation from leaky guts, persistent immune activation, immune dysregulation and CD4 T cell homeostasis failure are keys to the pathogenesis of HIV that leads to CD4 T cell depletion and immune compromise in HIV [25–27]. A summary of the host immune response following exposure to HIV is shown in Fig. 2.

**Table 1:** The comparison between SARS-CoV 2 and HIV characteristics

	SAR-CoV 2	HIV
Classification of virus	β coronaviruses	Lentiviruses
Virus size in diameter	60-140 nm	100 nm
Receptor binding domain	Angiotensin converting enzyme 2 (ACE2) receptor	CD4 T cell receptor and co-receptor CCR5, CXCR4
High expression of the receptor in human organ	lung, heart, ileum, kidney and bladder	Various lymphoid tissues
Primary affected cells	T cell lymphocytes	T cell lymphocytes
Immune activation	Acute cytokine storm	Chronic immune activation (with slow progressive immunodeficiency)
Transmission	Droplet, contact, airborne	Sexual transmission is the most common route, blood
Prevention	Mask, Social distancing	Condom use, Pre-exposure prophaylaxis (PrEP), and treatment as prevention (TasP)
Vaccine	As of 17 March 2021, 6 vaccines are authorized for emergency use based on preliminary evidence that they are safe and effective and 7 vaccines are approved for full use in few countries^*^	Unlikely up-to-now

* Available from https://www.nytimes.com/interactive/2020/science/coronavirus-vaccine-tracker.html

The lymphocyte analysis studies among patients with COVID-19 demonstrate that T lymphopenia—in particular, a decrease in CD4+ T cells—were common among patients with COVID-19, and more evident in severe cases [28, 29]. If superimposed on low CD4 counts in patients with advanced HIV disease, the lymphopenia of COVID-19 could delay the clearance of SARS-CoV-2 and promote disease progression. Moreover, COVID-19–associated pulmonary aspergillosis (CAPA) among severely ill COVID-19 patients has been reported worldwide, raising concerns of an additional contributing factor to mortality [30–32]. The pathological factors that may be associated with CAPA are the damaged respiratory epithelium, dysfunctional mucociliary clearance and low immune response, including low CD4 cell count [33, 34].

Elevated levels of multiple proinflammatory cytokines such as IL-2, IL-6, IL-10, and IFN-γ in severe cases of COVID-19 cannot only promote a hypercoagulable state, but also enhance the pro-thrombotic functions [35]. Moreover, a study reported a pattern of tissue damage consistent with complement-mediated microvascular injury among individuals with severe COVID-19 [36]. High incidence of thrombotic complications has been reported among COVID-19 patients admitted in intensive care unit [37].

HIV infection also is recognized as a prothrombotic condition [38–40]. Hence, COVID-19 and HIV co-infected patients could have a high risk of developing thrombosis. However, further studies are needed to prove this possibility.

## Clinical presentation and treatment outcome of COVID-19 among patients with HIV infection

Although several large cohort studies have reported the clinical characteristics and treatment outcome of COVID-19 in the general population [3, 4], clinical outcomes data on COVID-19 among patients with HIV are sparse. In this section, we summarize the demographics, clinical presentation and treatment outcome of COVID-19 among HIV-infected individuals from case series as well as epidemiological and cohort studies (Table 2).

**Table 2:** Summarization of HIV-infected individuals’ demographic data, clinical presentation and treatment outcome of COVID-19

	Hsi-en Ho, et al. [43]	Sigel et al. [49]	Vizcarra et al. [41]	Gervasoni et al. [54]	Meyerowitz et al. [55]	Shalev et al. [45]	Byrd et al. [52]	Okoh et al. [44]	Childs et al. [53]	Suwanwongse et al. [46]	Guo et al. [42]
No. of patients	93	88	51	47	36	31	27	27	18	9	8
Country	USA, New york	USA, New york	Spain	Italy	USA, Boston	USA, New york	USA, Rhode Island	USA, New Jersey	United Kingdom	USA, New york	China, Wuhan
Median age, Years (median, IQR)	58 (52–65)	61 (54–67)	53 (Mean; range 31–75)	51 ± 12 (Mean ± SD)	53 (Mean; range 24–81)	60.7 (Mean; range 23–89)	51 (40.5–57)	58 (50–67)	52 (49–58)	58 (52–70)	57 (48–62)
Male, n (%)	67 (72%)	66 (75%)	43 (84%)	36 (76%)	21 (58%)	24 (77%)	21 (78%)	15 (55%)	12 (67%)	7 (78%)	7 (88%)
Comorbidities, n (%)											
Hypertension	49(53%)	33 (38%)	18 (35%)	14 (30%)	11 (31%)	21 (68%)	6 (22%)	16 (59%)	6 (33%)	5 (56%)	NA
Diabetes mellitus	32(34%)	24 (27%)	7 (14%)	3 (6%)	8 (22%)	13 (42%)	4 (15%)	9 (33%)	4 (22%)	3 (33%)	NA
Chronic kidney disease	16(17%)	19 (22%)	6 (12%)	4 (9%)	8 (22%)	7 (23%)	1 (4%)	10 (27%)	5 (28%)	NA	NA
HIV clinical characteristics											
Time since HIV diagnosis, years, (median, IQR)	20 (15–26)^d^	NA	19.5 (9.3–28.6)	NA	NA	NA	10 (5.5–16.5)	NA	14.6 (9.7–23.4)	NA	NA
Preadmission CD4 count, cells/μLc (median, IQR)	554 (339–752)^e^	NA	565 (296–782)	636 ± 290 (Mean ± SD)	660 (382–949)	396 (Mean; range 89–924)^k^	644 (359–775)	551 (286–710)	395 (238–680)	465 (376–652)^o^	546 (295–709)
Preadmission HIV RNA < 200 copies/mL, n (%)	57 (84%)^f^	66 (81%)^l^	50 (98%)	44 (94%)	NA	30 (96.8%)^k^	27 (100%)	26 (96%)^i^	17 (94%)	8 (100%)^o^	8 (100%)
COVID-19 clinical presentations											
Time since onset of COVID-19 symptoms to admission or diagnosis, Days, median (IQR)	NA	NA	NA	NA	5.7 (Mean)	NA	5(2.5-8)^a^	NA	8 (7–10)	NA	NA
Presenting symptoms, n (%)											
Fever	61 (66%)	NA	36 (71%)	41	21 (58%)	23 (74%)	9 (33%)	17 (63%)	11 (61%)	5 (56%)	NA
Cough	71 (76%	NA	37 (73%)	23	20 (56%)	NA	6 (22%)	18 (67%)	13 (72%)	6 (67%)	NA
Shortness of breath or dyspnea	57 (61%)	NA	28 (55%)	10	14 (39%)	NA	9 (33%)	17 (63%)	12 (67%)	6 (67%)	NA
Infiltrates on radiograph, n (%)	71 (79%)^g^	NA	NA	12 (25%)^c^	NA	20 (65%)	13(48%)^b^	NA	13 (72%)	8 (89%)	NA
Needed Mechanical ventilation, n (%)	15(21%)	21%^m^	5 (10%)	2(4%)	7(19%)	8 (26%)	NA	NA	5 (28%)	5 (56%)	NA
COVID severity, n (%)	Most mild to moderate	Most mild to moderate	Mild or moderate: 38 (75%), Severe: 13 (25%)	Most mild to moderate	Most mild to moderate	Most severe (68%)	Most mild to moderate	Most mild to moderate	Most mild to moderate	Most severe	Most mild to moderate
Outcome of COVID-19, n (%)											
Cure or discharge	53 (74%)^h^	70(79%)^n^	44 (86%)	45(96%)	30(83%)	21 (68%)	26(96%)	25(85%)	12(67%)	2(22%)	NA
Hospitalized	0 (%)	NA	5 (10%)	NA	4 (11%)	2 (7%)	0 (0%)	NA	1 (5%)	0 (%)	NA
Died	19 (26%)^h^	18(21%)	2 (4%)	2 (4%)	2 (6%)	8 (26%)	1 (4%)	2 (15%)	5 (28%)	7 (78%)	1 (12.5%)

COVID-19: coronavirus disease 2019; HIV: human immunodeficiency viruses; ART: antiretroviral therapy; PI: Protease inhibitor; INSTI: Integrase inhibitors; NNRTI: Non-nucleoside reverse transcriptase inhibitor; NRTI: Nucleoside reverse transcriptase inhibitor

All of the patients in the studies that were shown in the Table 2 presented with symptoms of COVID-19

^a^Data available for analysis n = 19

^b^Data available for anlysis  n = 17

^c^Chest Computed Tomography-confirmed pneumonia

^d^Data available for analysis  n = 57

^e^Data available for analysis  n = 64

^f^Data available for analysis n = 68

^g^Data available for analysis n = 90

^h^Outcome in those hospitalized n = 72

^i^Viral load < 120 copies/ml

^k^HIV RNA level at admission

^l^HIV RNA level at admission or within previous 12 months (N = 82)

^m^Only percentage was reported

^n^Combined between discharge and still hospitalized

^o^Data available for analysis n = 8

Many studies conducted among PLWH with COVID-19 reported a median age range between 40 and 61 years and majority of them were men, which is similar to that reported in the general population [3, 4]. Most of the PLWH with COVID-19 were on antiretroviral therapy and were virologically suppressed. The incidence rate of COVID-19 infection among PLWH differs by country. The US (0.8%) [3] and Spain (1.8%) [41] reported an incidence based on PCR whereas China (0.68%) [42] used both PCR and clinical diagnosis. However, the rate of COVID-19 and HIV co-infection may be overestimated because PLWH are considered a high-risk population for developing complications and thus may be tested more frequently for COVID-19 compared to other populations. It has been reported that among COVID-19 and HIV co-infected patients, there is a high prevalence of comorbidities such as hypertension, diabetes and chronic kidney disease. The most common symptoms of COVID-19 detected were fever, cough or shortness of breath [43–46] which are similar to those reported in people without HIV [3, 47].

According to a case series, COVID-19-related mortality is high among immunosuppressed persons such as kidney transplant recipients and cancer patients [48]. There is a concern that COVID-19 disease may be more severe in persons with immunodeficiency or immune dysregulation. However, most PLWH infected with COVID-19 were reported to be on ART and had well controlled HIV infection. These people had suppressed HIV viral load and CD4 cell count > 350 cells/mm^3^. Previous studies have reported that nadir and recent CD4+ T cell counts or viral suppression preceding COVID-19 presentation were not associated with mortality [43, 49, 50]. No significant differences were reported in clinical characteristics, treatments, or outcomes between HIV individuals with recent CD4 counts < 200 or ≥ 200 cells per μL [41].

The demographic data, clinical presentation and treatment outcome among PLWH co-infected with COVID-19 are summarized in Table 2. Most of the PLWH co-infected with COVID-19 have had mild to moderate disease [41, 43, 44, 49, 51–56]. However, two studies reported that the majority of COVID-19 cases among PLWH were severe and associated with a high mortality rate [45, 46]. Additionally, risk factors for severe COVID-19 among PLWH were similar to those without HIV such as older age and comorbid medical conditions [42, 55].

## The impact of antiretroviral HIV drugs among patients co-infected with COVID-19

The S protein of the coronavirus mediates viral entry into targeted cells by binding to its receptor, ACE2, which leads eventually to viral fusion with the host cell membrane. However, the S protein is synthesized as an inactive precursor that requires activation by host cell proteases TMPRSS2 or cathepsin B or L to become a fusion active form. Thus, protease inhibitors have been considered as a candidate therapy for SARS-CoV and other coronaviruses [57, 58]. Treatment with the HIV protease inhibitor with lopinavir/ritonavir (LPV/r) showed some benefit in a non-human primate model of MERS-CoV infection [59]. Recent in vitro studies reported that antiviral activity of LPV/r against SARS-CoV-2, albeit at a relatively high EC_50_ [60, 61]. However, several observational studies and case reports demonstrated that the clinical benefit of using LPV/r among COVID-19 patients was inconclusive [62–65]. Moreover, a randomized, controlled open-label trial conducted in hospitalized adult patients with severe COVID-19 failed to show a statistically significant difference in time to clinical improvement or mortality in the LPV/r-treated group as compared to standard of care [66]. Moreover, LPV/r treatment was stopped early in 13 patients (13.8%) because of adverse events. The lack of clinical benefit of LPV/r for COVID-19 was confirmed in the RECOVERY and SOLIDARITY trials [67, 68].

In vitro data show the absence of any anti-SARS-CoV-2 activity of darunavir [69]. This finding is corroborated by the clinical observation that PLWH receiving a darunavir-containing regimen were not protected from COVID-19 [70].

Antiviral activity of tenofovir against SARS-CoV-2 has been demonstrated in virtual and in vitro studies [71, 72]. These results led to speculation that tenofovir disoproxil fumarate (TDF)– and tenofovir alafenamide (TAF)–containing antiretroviral regimens might have a protective effect against COVID-19 [3]. However, a prospective cohort in Spain observed a higher rate of COVID-19 infection among PLWH on TAF or TDF [41]. This finding supports the results from case series that tenofovir-based ART does not provide any clinical benefit against COVID-19 among PLWH [45, 52]. Therefore, up until now, the use of antiretroviral agents has no clinical benefit for the treatment or prevention of COVID-19.

## HIV service during COVID-19 outbreak

COVID-19 pandemic has had an unprecedented negative impact on HIV services and care across the globe. According to a WHO report, between April and June 2020 73 countries faced the risk of ART disruption affecting 17.7 million people receiving ART [9]. A modelling study done by WHO and UNAIDS estimated that a 6-month disruption of ART could lead to more than 500,000 extra deaths from AIDS-related illnesses in sub-Saharan Africa in 2020–2021 [73].

Several factors have contributed to the disruptions in HIV service and care during the COVID-19 pandemic. First, the strict quarantine measures and transportation lock downs were implemented in many cities to control the spread of COVID-19. A survey from China reported that PLWH who returned to their home towns could not refill their medication due to the lock down [74]. Not surprisingly, the shortage of ART affected not only the physical health status of PLWH but also caused considerable psychological stress. Postal delivery of drugs is one approach to ensure that PLWH have a continuous supply of ARVs. However, this method poses implementation challenges because not all PLWH want ARVs delivered to their homes in order to maintain privacy and guard against unwanted disclosure of their HIV status to household members [75]. Second, there have been shortages of ARV because of shut downs by certain drug manufacturers [9]. Third, healthcare workers who provide care for PLWH were diverted to care for patients with COVID-19 [75–77]. A survey performed in Central and Eastern Europe reported that infectious disease physicians shifted their focus to take care COVID-19 patients instead of PLWH [78].

During this difficult time, healthcare systems must develop effective strategies for balancing healthcare resources need by both PLWH and patients with COVID-19. ARV multi month dispensing (MMD) policy has been adopted in many countries [9] in order to prevent disruptions of ARV supplies for PLWH and to reduce their exposure to COVID-19 when accessing HIV services. Telemedicine platforms have been proposed as a strategy for continued provision of healthcare to PLWH in response to COVID-19 during the lock-down period and have been implemented successfully in many settings [79]. A multidisciplinary approach to help PLWH maintain their physical and mental health has never been more important than now, in the face of the COVID-19 pandemic. In addition, other services for prevention and treatment of opportunistic infection and sexually transmitted infections may be disrupted during COVID-19 pandemic. This lockdown related disruptions may increase in burden of TB and sexually transmitted infections [80–82].

## Conclusion

Even though lymphopenia associated with COVID-19 may further decrease CD4+ T cell counts in PLWH, there are no differences in the clinical presentations, outcomes, morbidity and mortality between individuals who have SARS-CoV-2 with or without HIV infection. Several randomized control trials have shown that antiretroviral therapy has no beneficial effect among people infected with SARS-CoV-2 compared to standard of care.

To date, we still do not have any proven antiviral agents that reduce mortality among COVID-19 patients. Right now, the medical resources have shifted towards COVID-19 but it should be noted that we should not overlook the care for PLWH who still need ART and follow-up care. We should use several innovative service deliveries and MMD policy to help PLWH have continuous supply of ARV during this outbreak.

## Data Availability

Not applicable.

## Abbreviations

HIV: Human immunodeficiency viruses
COVID-19: Coronavirus disease 2019
SARS-CoV-2: Severe acute respiratory syndrome coronavirus 2
WHO: World Health Organization
PLWH: People living with HIV
ART: Antiretroviral therapy
ACE2: Angiotensin converting enzyme 2
APC: Antigen presenting cells
DCs: Dendritic cells
ARDS: Acute respiratory distress syndrome
CCR5: Chemokine receptor 5
CXCR4: Chemokine receptor 4
LPV/r: Lopinavir/ritonavir
TDF: Tenofovir disoproxil fumarate
TAF: Tenofovir alafenamide
MMD: Multi month dispensing
